# B lymphocyte stimulator modulates number and function of endothelial progenitor cells in systemic lupus erythematosus

**DOI:** 10.1186/s13075-019-2015-7

**Published:** 2019-11-21

**Authors:** Francesca Romana Spinelli, Cristiana Barbati, Fulvia Cecarelli, Francesca Morello, Tania Colasanti, Marta Vomero, Laura Massaro, Valeria Orefice, Cristiano Alessandri, Guido Valesini, Fabrizio Conti

**Affiliations:** Department of Internal Medicine and Medical Specialties, Rheumatology, Sapienza University of Rome, Rome, Italy

**Keywords:** Systemic lupus erythematosus, Endothelial progenitor cells, Atherosclerosis, BLyS, Belimumab

## Abstract

**Background:**

Circulating endothelial progenitor cells (EPCs) are biologic markers of endothelial function. In patients with systemic lupus erythematosus (SLE), the numerical reduction and functional impairment of EPCs contribute to the endothelial dysfunction.

Through ex vivo and in vitro studies, we aimed at evaluating the effects of B lymphocyte stimulator (BLyS) on EPC colonies and endothelial cells and also investigating BLyS receptor expression on these cells.

**Methods:**

EPCs were isolated from peripheral blood mononuclear cells (PBMC). In order to evaluate their ability to form colonies, EPCs were cultured on fibronectin-coated dishes and incubated with BlyS alone or BlyS and belimumab. Apoptosis of EPCs and endothelial cell line EA.hy926 was evaluated after 6, 12, and 24 h of incubation with BLyS and after 6 h with BLyS and belimumab. The expression of B cell activating factor-receptor (BAFF-R), B cell maturation antigen (BCMA), and transmembrane activator and calcium modulator and cyclophilin ligand (CAML) interactor (TACI) on EPCs and EA.hy926 was analyzed by cytofluorimetry.

**Results:**

The number of EPC colonies was lower in patients than in controls. Moreover, the colonies from SLE patients were poorly organized compared to controls; the addition of belimumab restored the colony structure. Incubation with BLyS induced apoptosis of EPCs and EA.hy926 that was inhibited by the co-incubation with belimumab. BAFF-R and BCMA were expressed on both EPCs and EA.hy926, while TACI was expressed only on EPCs.

**Conclusions:**

EPCs and endothelial cells preferentially express BAFF-R which could be involved in the pro-apoptotic effect of BlyS. Belimumab administration seems to restore the quantitative and qualitative changes of EPC colonies both ex vivo and in vitro**.**

## Background

Circulating endothelial progenitor cells (EPCs) are bone marrow-derived precursors which differentiate into mature endothelial cells and contribute to new vessel formation and vascular homeostasis [1]. Circulating progenitors are crucial for the vascular damage repair and their impairment is associated with increased subclinical atherosclerosis [2]. Traditional cardiovascular risk factors such as hypertension, diabetes, dyslipidemia, and smoking habit affect both the number and functions of EPCs [3]. Besides the traditional risk factors, chronic inflammatory diseases, such as rheumatoid arthritis (RA), are associated with an impairment of endothelial progenitors [4].

The increased risk of cardiovascular (CV) morbidity in patients with systemic lupus erythematosus (SLE) is mostly driven by disease-related risk factors during the early stages of the disease and the occurrence of traditional risk factors later on [5, 6]. In SLE patients, the increased vascular damage is accompanied by an inadequate vascular repair leading to endothelial dysfunction. Several studies suggested that numerical and functional impairment of EPCs detected in SLE patients contribute to the endothelial dysfunction [7–11]. Type I IFN, a cytokine playing a key role in the pathogenesis of SLE, contributes to EPC dysfunction promoting abnormal angiogenesis, boosted by an excess of IL10 [7, 12]. B lymphocyte stimulator (BLyS)—also named B cell activating factor (BAFF)—is a B cell survival factor which increases in the serum of SLE patients and correlates with disease activity [13]. BLyS exerts its action through three different receptors: BAFF-R, B cell maturation antigen (BCMA), and transmembrane activator and calcium modulator, and cyclophilin ligand (CAML) interactor (TACI) [13]. The three receptors are expressed on B cells, but also on T cells and dendritic cells [13]. Recent experimental evidence could suggest a role of BLyS in atherosclerosis: indeed, BAFF-R-deficient mice, as well as in mice treated with an anti-BAFF monoclonal antibody, experienced a reduction of the atherosclerotic plaque size [14]. However, data in humans are still lacking and, to the best of our knowledge, no previous studies investigated the effect of BLyS on endothelial progenitor cells and mature endothelial cells.

Aim of this study was to assess the effect of BLyS and its inhibition on number and function of EPC both in vitro and ex vivo in SLE patients. Furthermore, we aimed at investigating the possible expression of BLyS receptors on EPC and mature endothelial cells.

## Methods

### Patients

We enrolled consecutive patients fulfilling at least four of the American College of Rheumatology (ACR) 1997 revised criteria for SLE [15] candidate to belimumab (anti-BLyS monoclonal antibody) for unresponsiveness to standard therapy. As required by the Italian regulation, all patients starting belimumab should be “serologically active” (positive for serum anti-dsDNA antibodies either by Farr assay, indirect immunofluorescence (IIF), or enzyme-linked immunosorbent assay (ELISA), and low C3 or C4 serum levels) and should have an active disease. Patients with previous cardiovascular events or impaired kidney function were excluded. As control, 8 age and sex-matched healthy subjects were studied. All patients signed an informed consent before entering this study. The protocol was approved by the Local Ethical Committee (Prot. 120/16). Demographic, clinical data and comorbidities were recorded at the baseline visit.

### Disease activity assessment

At each visit (baseline, after 4 and 12 weeks of belimumab treatment), SLE disease activity was evaluated by Systemic Lupus Erythematosus Disease Activity Index (SLEDAI) 2K.

### Blood sample collection

Blood samples were collected from each patient at baseline and after 4 and 12 weeks of belimumab. The cells were isolated on the same day of the blood draw. As for the control group, blood samples from healthy subjects were collected on the same day of baseline patients’ visit.

### Quantification of circulating endothelial progenitor cells by flow cytometry

Peripheral blood mononuclear cells (PBMCs) were obtained by density gradient centrifugation (Lympholyte-H; Cedarlane Laboratories, Hornby, Ontario, Canada); phenotypic characterization was performed as previously described by Vasa et al. [16]. In brief, after incubation with FcR-blocking reagent (Miltenyi Biotec, Bergisch-Gladbach, Germany), 1 × 10^6^ PBMCs were incubated for 30 min on ice with fluorescein isothiocyanate (FITC)-labeled mAb anti-CD34 (BD Immunocytometry Systems, San Jose, CA, USA) and phycoerythrin (PE)-labeled mAb anti VEGF R2/KDR (BD Immunocytometry Systems, San Jose, CA, USA). Appropriate isotype controls were used. Acquisition was performed on a FACS Calibur (BD Immunocytometry Systems, San Jose, CA, USA) and included 100,000 to 400,000 events per sample. Data were analyzed using the CellQuest Pro software (BD Immunocytometry Systems; San Jose, CA, USA). EPCs were defined as CD34/KDR double-positive cells, and their number was expressed as a percentage of cells within the lymphocyte gate.

### Cell cultures and colony-forming unit assay

For the isolation of EPCs, we used 20 ml of venous blood. Samples were processed within 4 h of collection, and peripheral blood mononuclear cells were isolated by Ficoll density-gradient centrifugation; 5 × 10^6^ PBMCs were plated on human fibronectin-precoated (10 μg/ml Sigma-Aldrich, six-well plates), cultivated in growth medium 199 (Gibco) containing 20% fetal bovine serum (FBS) and penicillin (100 U/mL)/streptomycin (100 μg/mL, Gibco), and then incubated (37 °C and 5% CO_2_). After 3 days, non-adherent cells were removed and fresh culture medium was supplied and changed every other day. After 7 days of culture, EPC colony-forming units (CFU) were enumerated by light microscopy (Scpe.A1, ZEISS). The ability to form colonies was evaluated after in vitro treatment of EPCs from HD with BLyS at 20 ng/ml alone or with belimumab at 173 μg/ml. An immortalized line of endothelial cells, human umbilical vein cell line *EA*.*hy926*, was also cultured in Dulbecco’s modified Eagle’s medium containing 10% FBS, 50 IU/ml penicillin, 50 μg/ml streptomycin, and 2 mM l-glutamine.

### Phenotypic characterization of cultured EPC

By day 7, most of the adherent cells had acquired a spindle shape, typical of early EPCs; flow cytometry analysis with CD34 FITC/KDR PE was performed.

### Apoptosis of endothelial progenitor cells and endothelial cell line

Endothelial progenitor cells and human endothelial cell *EA.hy926* were incubated with BLyS at concentrations of 5 and 20 ng/ml; apoptosis was investigated after 6, 24, and 48 h of treatment and re-evaluated after 6 h of co-incubation with belimumab at 173 and 313 μg/ml.

The percentage of apoptotic cells was evaluated using annexin V (AV) and propidium iodide (PI) apoptosis detection kit (MBL) by flow cytometry analysis**.** Acquisition was performed on a FACS Calibur and included 10,000 events.

### Evaluation of BLyS receptors on EPC and *EA.hy926* cell surface

After pre-incubation with an FcR-blocking reagent, cells were labeled for 30 min on ice with mAb anti-BAFF-R FITC, anti-BCMA PE, and anti TACI APC (Biolegend, San Diego, CA, USA). As positive control for the expression level of BLyS receptors, we used B lymphocytes labeled with mAb anti-CD19 and anti-BLyS receptor mAbs as described above. Appropriate isotype controls were used. Acquisition was performed on a FACS Calibur and included 10,000 events for apoptosis and 50,000 events for BLyS receptors. Data were analyzed using the CellQuest Pro software (BD Immunocytometry Systems; San Jose, CA, USA); the results were expressed as mean fluorescence intensity (MFI).

### Statistical analysis

Statistical evaluations were performed using GraphPad Prism Version 6 (GraphPad Software, San Diego, CA, USA). Data were expressed as mean + standard (SD) deviation or median (IQR) depending on the variable’s distribution, and parametric or non-parametric tests were used accordingly. ANOVA test was used to compare different populations. *P* values ≤ 0.05 were considered significant.

## Results

### Patients

We enrolled 18 Caucasian female patients [mean age 45.0 ± 9.5 years, mean disease duration 18.3 ± 10.7 years] with active disease [median baseline SLEDAI 6 [4]]. None of the patients had concomitant anti-phospholipid syndrome or anti-phospholipid antibody positivity. The mean weekly prednisone dose at baseline was 65 + 16.6 mg and did not significantly change at 4 and 12 weeks. The concomitant therapies remained stable throughout the study period. Table 1 summarizes the clinical features of the cohort. Belimumab was administered intravenously at 10 mg/kg at baseline and after 2, 4, 8, and 12 weeks.

**Table 1:** Clinical features and concomitant treatment of the cohort

	SLE patients (*n* = 18)
Baseline SLEDAI, median (IQR)	6 (4)
Follow-up SLEDAI, median (IQR)	5 (2)*
Indication for starting belimumab	
Musculoskeletal involvement, *n* (%)	12 (66.7)
Mucocutaneous involvement, *n* (%)	5 (27.8)
Lung involvement, *n* (%)	1 (5.5)
Concomitant treatment	
Glucocorticoids, *n* (%)	18 (100)
Hydroxychloroquine, *n* (%)	17 (94.4)
Mycophenolate, *n* (%)	5 (27.8)
Azathioprine, *n* (%)	4 (22.2)
Cyclosporine, *n* (%)	3 (16.7)
Methotrexate, *n* (%)	2 (11.1)
Thalidomide, *n* (%)	1 (5.5)

*p = 0.001 vs baseline

### Number of circulating EPCs

**Fig. 1:**
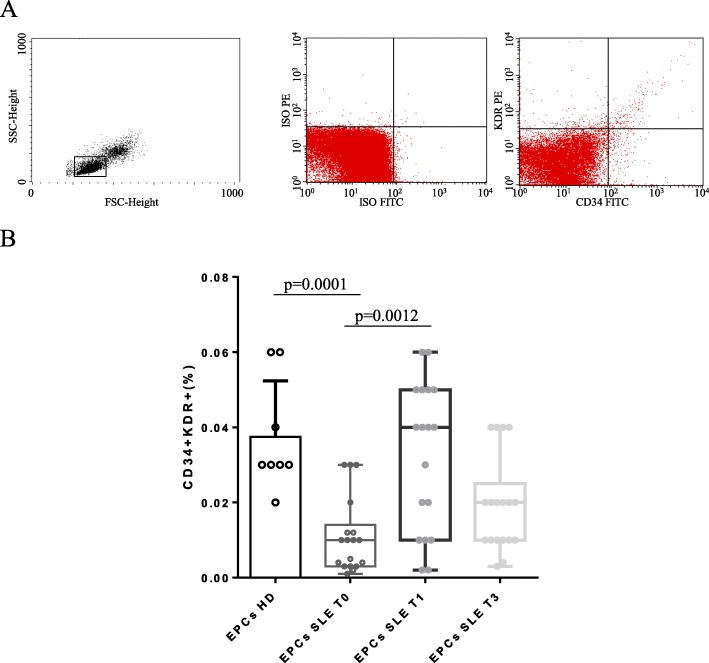
Flow cytometry analysis of CD34+KDR+ cells and percentage of circulating EPCs. **a** Representative flow cytometry histogram plots obtained from HD are shown. Left panel, forward scatter (FSC) and side scatter (SSC) with lymphocyte gate are indicated. Right panel, double fluorescence with FITC-labeled CD34 and PE-labeled KDR antibodies are reported. In the top right quadrant, CD34/KDR double-positive cells are indicated. Quadrants are set on the basis of isotype controls in the middle panel. **b** Histogram shows the mean percentage of EPCs from SLE patients at baseline (T0) and after 1 and 3 months (T1 and T3) of treatment with belimumab and HD

EPCs were defined as CD34/KDR double-positive cells, and their number was expressed as a percentage of cells within the lymphocyte gate, as shown in Fig. 1a. The EPC number was significantly lower in SLE patients than in HD (*p* = 0.0001). At week 4 (T1), after two administrations of belimumab, median EPC number significantly increased from 0.010 (0.011)% to 0.04 (0.05)% (*p* = 0,0012 vs SLE T0). After 12 weeks (T3), EPC number did not significantly differ compared to 1 month, nor to baseline (Fig. 1b).

### Effect of BLyS and belimumab on colony-forming units

**Fig. 2:**
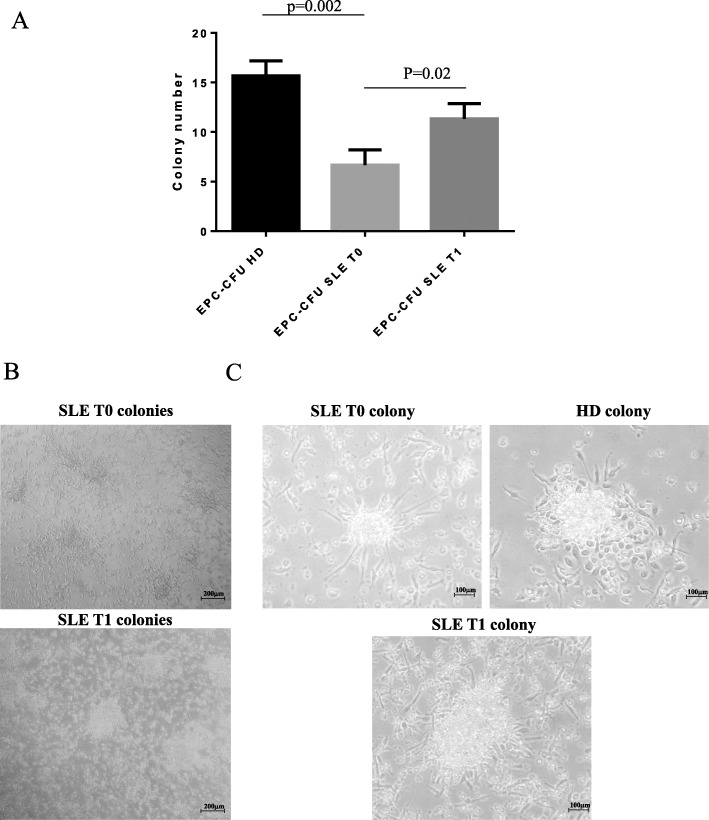
Number and morphology of EPC-CFU. **a** Histogram shows the mean number of EPC-CFU from HD and SLE at baseline (T0) and 1 month (T1) later belimumab. **b** The panels show the difference in number and organization between EPC-CFU from SLE to T0 and T1 at lower microscopic magnification. **c** Figures are representative of the morphology of EPC-CFU from SLE patients at T0 and T1 and HD. CFU from HD (top right panel) has a typical morphology of EPC-CFU, while CFU from SLE at T0 (top left panel) appears small and disorganized. CFU from SLE at T1 seems to be similar to those from HD in morphology and organization, as shown in the bottom panel. Images obtained in a representative experiment are shown

At baseline, the number of EPCs-CFU from SLE patients was significantly lower compared to HD (6.7 + 1.5 vs 15.67 + 1.5, *p* = 0.02, Fig. 2a). After 4 weeks of treatment with belimumab, EPCs-CFU significantly increased compared to T0 (11.33 + 0.88 vs 6.7 + 1.5, *p* = 0.03, Fig. 2a).

The lower microscopic magnification shows the morphological differences of EPCs-CFU of SLE patients between T0 and T1 (Fig. 2b). As shown in Fig. 2c, the morphology of the individual colonies changed radically: the colonies from SLE patients were smaller and less organized than those from HD. At T1, the colonies became more organized, resembling those of HD.

**Fig. 3:**
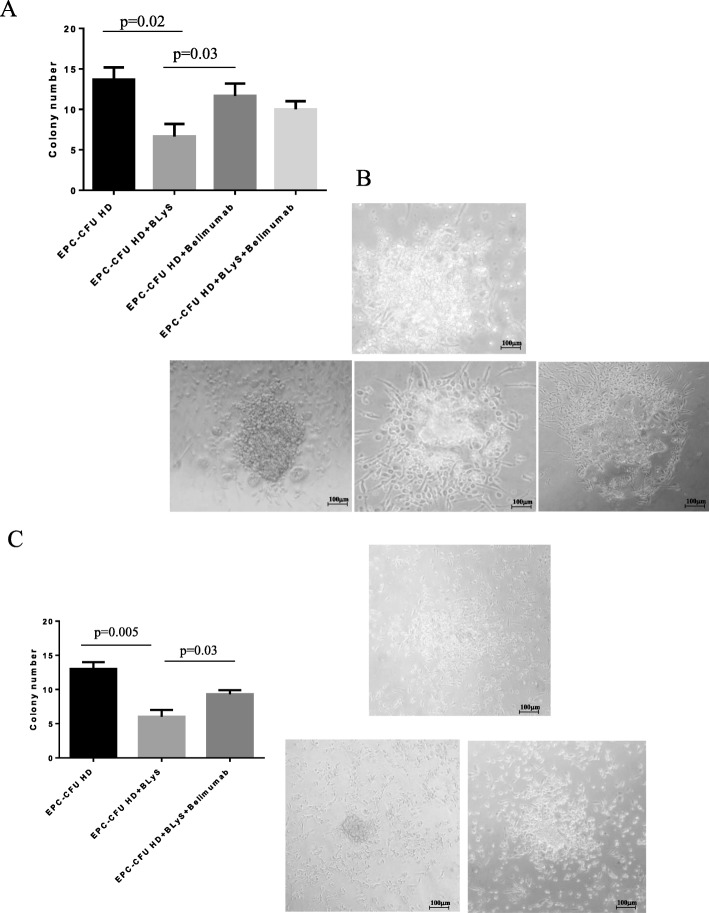
In vitro effect of BLyS and belimumab on EPC-CFU number and morphology. **a** Histogram shows the mean number of EPC-CFU reached after treatment of EPCs from HD with BLyS/belimumab alone or in co-treatment at day 7 of the culture. **b** Images show the change of morphology of EPC-CFU in vitro treated with BLyS that appears disorganized (low left panel), while co-treatment with belimumab seems to preserve a morphology like a normal colony of EPCs (low right panel). Belimumab does not affect CFU morphology (low middle panel) that is comparable to that of HD (top panel). Images obtained in a representative experiment are shown. **c** Histogram shows the mean number of EPC-CFU reached after treatment of EPCs with BLyS alone or with belimumab at the start of the culture. EPCs treated with BLyS are not able to form a normal colony, as shown in the low left panel, while co-treatment with belimumab (low right panel) leads the cells to create a colony comparable with that observed in untreated cells (top panel). Images obtained in a representative experiment are shown

At day 7, EPCs from HD were incubated with BLyS at 20 ng/ml for 6 h. Treatment with BLyS significantly decreased the number of colonies (6 + 1.5 vs 12.67 + 1.1 in treated and untreated cultures, respectively). Belimumab alone had no effect on EPC colonies; however, the addition of belimumab inhibits the effect of BLyS on the number of colonies (10 + 1.1 vs 6 + 1.5) (Fig. 3a).

We observed a substantial change in the CFU morphology of BLyS-treated cells; the addition of belimumab at 173 μg/ml reverted the effect of BLyS (Fig. 3b).

When we added BLyS at day 0 to the freshly isolated EPCs, after 7 days, the number of colonies was lower compared to untreated cells (6.7 + 0.8 vs 13.67 + 0.8, *p* = 0.005); the co-treatment with BLyS and belimumab partially inhibited this effect, as showed in Fig. 3c (10 + 0.5 vs 6.7 + 0.8, *p* = 0.03). Additionally, the colony morphology showed significant differences between the groups: the cells treated with BLyS were not able to form an organized and homogeneous colony like those of HD untreated cells and belimumab co-treated cells (Fig. 3c).

### Phenotypic characterization of cultured EPC

**Fig. 4:**
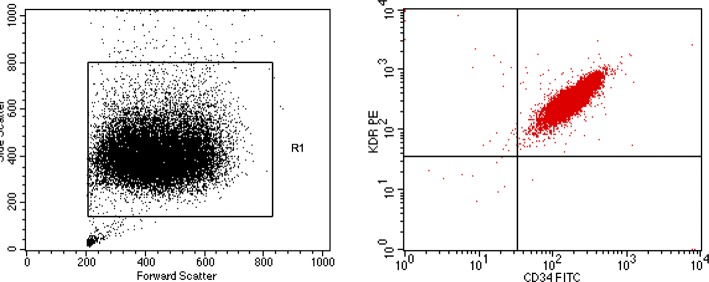
Phenotypic characterization of cultured EPCs at day 7 by showing the expression of CD34 and KDR. Cytofluorimetric images are gating from EPC population (left top panel); 100% of EPCs express CD34/KDR, as shown in the right panel

At day 7, we characterized the expression of CD34, KDR, and CD45 by flow cytometry. As showed in Fig. 4, the totality (100%) of EPCs expressed CD34 and KDR [17].

### Apoptosis of endothelial progenitor cells and EA.hy926 endothelial cells (EC)

**Fig. 5:**
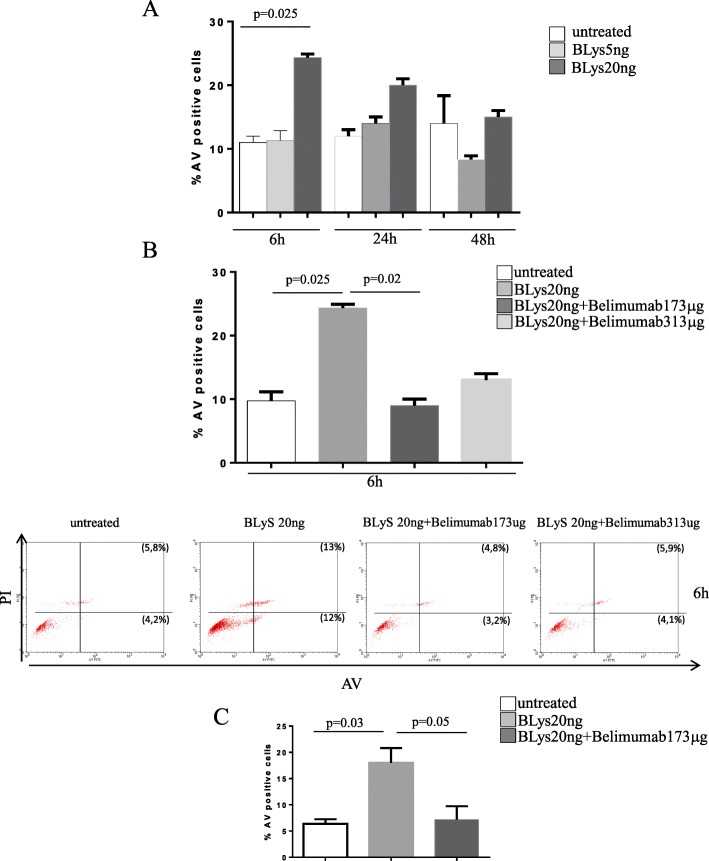
Effect of BLyS and belimumab on EPC and *EA.hy926* apoptosis. **a** Histograms show the potential effect of BLyS on EPC apoptosis induction. **b** Histogram reports the protective role of belimumab against the effect of BLyS on EPC apoptosis. Bottom panels are dot plots representative of three distinct experiments. **c** Histogram shows the potential effect of BLyS on *EA.hy926* apoptosis and the protective effect of belimumab

We investigated the apoptotic potential of BLyS by treating EPCs with different BLyS concentrations (Fig. 5a). To better evaluate BLyS-induced cell death, we performed a time-dependent analysis of apoptosis. We cultured EPCs in the presence of BLyS at different time points (6, 24, and 48 h). A significant increase of BLyS-induced apoptosis was already detectable at 6 h (24.33 + 0.6% vs untreated cells, *p* = 0.025), reaching a plateau at 24 h (Fig. 4a). The addition of belimumab inhibited the apoptotic effect of BLyS both at 173 and 313 μg/ml (9 + 1.4% and 13 + 0.5% vs BLyS-treated cells, respectively) (Fig. 5b). Belimumab alone had no effect on cell fate (data not shown). We selected the dose of 20 ng/ml and 173 μg/ml as optimal BLyS and belimumab concentration respectively, for in vitro studies on *EA.hy926* cell apoptosis and EPCs-CFU capacity (as described above). BLyS induced apoptosis of *EA.hy92*, and co-treatment with belimumab inhibited the effect (18 + 2% and 7 + 1.8% vs untreated cells, *p* = 0.03 and *p* = 0.05, respectively) (Fig. 5c).

### Expression of BAFF-R, BCMA, and TACI on EPC and EC surface

**Fig. 6:**
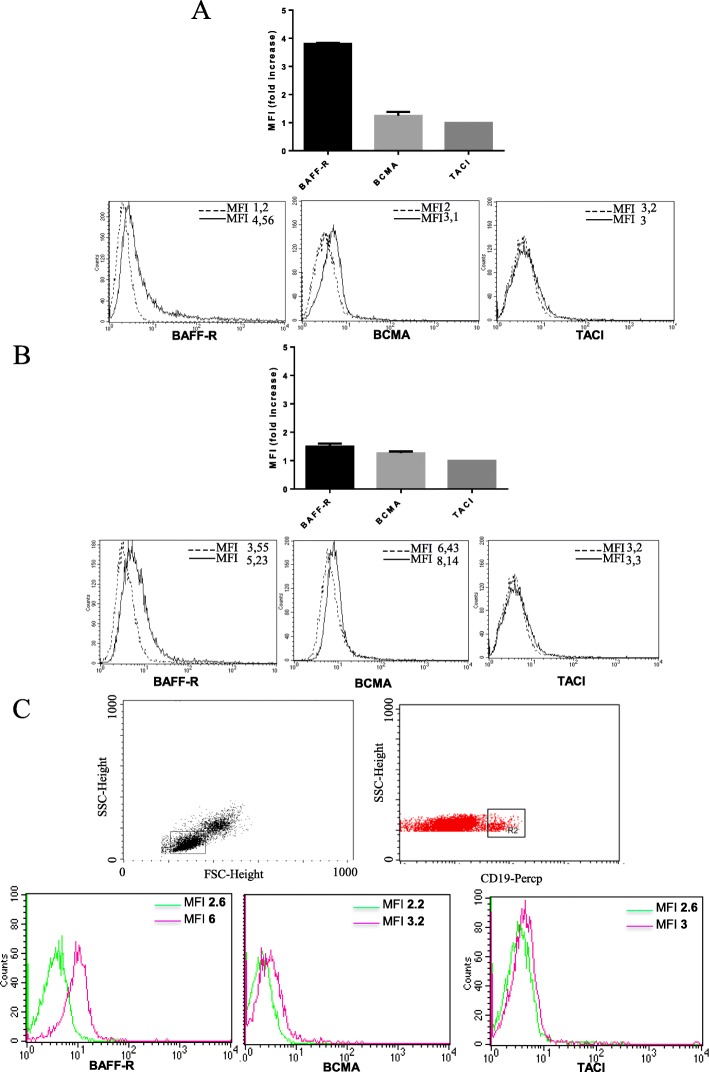
Expression of BAFF-R, BCMA, and TACI on EPC and *EA.hy926* surface. **a** Flow cytometry analysis after staining of EPCs in vitro cultured with anti-BLyS receptors mAbs: BAFF-R, BCMA, and TACI (left, middle, and right panels, respectively). Isotype control staining is represented by the broken line and anti-BLyS receptors by the black-solid line. Results obtained in a representative experiment are shown. **b** Flow cytometry analysis after staining of *EA.hy926* with anti-BLyS receptors mAbs: BAFF-R, BCMA, an TACI (left, middle, and right panels, respectively). Isotype control staining is represented by the broken line and anti-BLyS receptors by the black-solid line. Results obtained in a representative experiment are shown. **c** Histrogram plots show CD19-positive cell stained for BLyS receptors: BAFF-R, BCMA, and TACI (left, middle, and right panels, respectively)

Finally, we analyzed the expression of BLyS receptors on the surface of angiogenetic cells. BAFF-R and BCMA, but not TACI, were expressed both on EPCs (Fig. 6a) and on *EA.hy926* cells (Fig. 6b). Moreover, the cytofluorimetric analysis for the characterization of the BLyS receptors on B cells confirms the functionality of the antibodies used. According to data reported in literature, our results showed that B lymphocytes preferentially express BAFF-R compared to BCMA, while TACI is weakly expressed (Fig. 6c).

## Discussion

The results of our study suggest that endothelial progenitor cells and endothelial cell line *EA.hy926* express BLyS receptors which may be involved in the angiogenic cell apoptosis. Treatment with belimumab inhibits the pro-apoptotic effect of BLyS increasing the number of circulating progenitor cells and improving their ability to form colonies.

Since the late 1980s, atherosclerosis is considered an immune-mediated disease which starts with the endothelial damage induced by humoral and cellular arms of innate and adaptive immune responses. Endothelial dysfunction, the earliest and reversible stage of atherosclerotic process, is widely documented in SLE patients and provides diagnostic and prognostic data on atherosclerotic risk [11]. Besides the ultrasonographic assessment of endothelial function, biomarkers of inflammatory, procoagulant, and oxidative status of endothelial cells provide information on endothelial health [18]. EPCs are immature precursors of endothelial cells, recruited from bone marrow, mirroring the reparative capacity of healthy endothelium; therefore, alterations of number and functions of EPCs reflect endothelial dysfunction [18]. The impairment of EPCs’ ability to repair the damaged endothelium contributes to the onset of endothelial dysfunction.

In the last years, several studies investigated the number and functions of EPCs in SLE patients. In agreement with most of the previous studies, we found a significantly reduced number of progenitor cells in peripheral blood of patients with SLE compared to healthy subjects [8–10, 19–21]. Only few studies detected a normal number of EPCs; however, these authors identified progenitor cells by cell markers other than CD34 and KDR used in our study [22, 23]. CD34 and KDR belong to hematopoietic stem cell and endothelial cell lines, respectively, whereas CD133 characterizes more immature progenitor cells, just released by bone marrow stromal niches [24]. Therefore, different stages of EPC maturation may account for the discrepancy in the results of previous studies. Using many combinations of cell markers, two studies demonstrated that only mature EPCs, represented by the CD34+/KDR+ population, are depleted in SLE patients, while the number of immature CD133-positive cells seems to be comparable with healthy subjects [19, 20].

Data on EPC functions are more concordant, showing reduced proliferative, migratory, and adhesive properties [7, 9, 19, 22, 23].

Traditional cardiovascular risk factors are known to affect EPC number and functions, both in the general population and in SLE patients [3, 25]. Most of our patients had one or more traditional risk factors that were stable between baseline and follow-up. Moreover, none of the patients presented uncontrolled hypertension or diabetes nor a definite metabolic syndrome that has been recently associated to a decrease of EPC in SLE patients [21]. The association with disease activity is less evident, and most of the previous studies did not detect any association between EPC number and SLE-related factors, including disease activity [25]. Compared to previous studies, we recruited patients with active disease, candidate to biological drugs; this may contribute to the very low number of EPCs detected in our cohort, even if the correlation between number of progenitors and disease activity at baseline was not statistically significant. Conversely, we found an association between the extent of the SLEDAI reduction and the number of EPCs. The role of immunomodulatory drugs on EPC number and functions is still unclear. In the study by Lee et al., EPCs were reduced in SLE patients irrespective of steroids, antimalarials, or cytotoxic drug use [8]. Westerweel et al. studied patients on hydroxychloroquine treatment and showed a significant higher number of EPCs [10]. On the contrary, Grisar et al. observed a reduction of EPCs in patients receiving antimalarials and also confirmed this observation in vitro, demonstrating a significant decrease of colony-forming units when chloroquine was added to EPCs [22]. In our study, we can exclude any effect of the treatment since all the drugs remained stable during the 12 weeks of follow-up.

In 2007, two separate groups of researchers suggested an intriguing link between IFNα, a key cytokine in SLE pathogenesis, and the reduction of endothelial progenitors [7, 8, 26]. Both studies suggested an association between EPC depletion and the so-called IFN signature demonstrating that EPCs were reduced especially in those patients presenting with more pronounced expression of IFN-related proteins [7, 8]. More recently, Kahlenberg et al. suggested a role for inflammasome activation—as demonstrated by the increase in caspase-1 and IL18—in the detrimental effect exerted by IFN on EPC [27].

The role of IFN in EPC impairment is particularly interesting since this cytokine is one of the emerging targets for SLE treatment. Currently, the only biological drug approved for SLE is belimumab, a fully human IgG1λ targeting BLyS. In this study, we evaluated ex vivo the effect of BLyS inhibition on EPC number demonstrating a rapid and significant increase of EPC number after short-term (4 weeks, 2 infusions) administration of belimumab.

Recently, the effect of B cell depletion therapies on atherosclerotic plaque in mice models outshined on the role of B lymphocytes in atherosclerosis. BLyS inhibition slowed the progression and reduced the size of the plaque in two different murine models of atherosclerosis [28, 29]. Whether BLyS may act even on endothelial activation is not yet known. Anti-BLyS could act by selectively depleting B2 cells, a sub-population of B lymphocytes with pro-atherogenic properties, or indirectly by reducing other pro-inflammatory cytokines [29]. A very recent study demonstrated a significant association between high levels of BLyS and genetic variants with subclinical atherosclerosis in SLE patients [30]. Moreover, in a recent study on lupus nephritis, the authors demonstrated the glomerular and tubulointerstitial expression of BLyS and TACI in proliferative lupus nephritis, thus suggesting an endothelial expression of the cytokine [31].

Starting from the clinical observation of the increase in the number of EPCs after administration of belimumab, we investigated whether the effect of BLyS on EPCs could be directly attributed to the cytokine rather than to the effect on disease activity. When we treated the EPCs and the endothelial cell line with BLyS, we detected a pro-apoptotic effect of the cytokine on both cell populations. Lee et al. proved that type I IFN induces apoptosis of the progenitor cells and that EPCs express IFNα/β receptors 1 and 2 [8]. Similarly, we observed that the endothelial cell line *EA.hy926* and, more markedly, the late EPC population express BAFF-R on their surface. BLyS receptors are expressed on many cell types—including not only B cells but also T lymphocyte and myeloid cells; however, to the best of our knowledge, their expression on angiogenic cells was never investigated before [13]. This evidence supports a dual—direct and indirect—role for BLyS in the atherosclerotic process.

This study has some limitations. Since belimumab has been administered to patients with active disease (as per local regulations), none of the subjects were treatment-naïve. However, the concomitant therapy remained unchanged from baseline to follow-up. Moreover, the short-term follow-up does not allow to draw any conclusion about the long-term prevention of cardiovascular events which, conversely, would require a long-term follow-up.

In summary, besides confirming a numerical and functional impairment of EPCs in SLE patients, we observed for the first time the expression of receptors for BLyS on angiogenic cells. The results of our study could suggest a direct role of BLyS on angiogenic cell apoptosis and its contribution to the imbalance between endothelial damage and repair. Besides decreasing the disease activity, belimumab could limit the progression of SLE-related atherosclerotic process by neutralizing the binding of BLyS to its receptors on angiogenic cells.

## Data Availability

The datasets used and/or analyzed during the present study are available from the corresponding author on reasonable request.

## Abbreviations

BAFF: B cell activating factor
BCMA: B cell maturation antigen
BLyS: B lymphocyte stimulator
CFU: Colony-forming units
EPCs: Endothelial progenitor cells
PBMCs: Peripheral blood mononuclear cells
TACI: Transmembrane activator and calcium modulator and cyclophilin ligand (CAML) interactor
